# Machine Learning Algorithms Evaluate Immune Response to Novel *Mycobacterium tuberculosis* Antigens for Diagnosis of Tuberculosis

**DOI:** 10.3389/fcimb.2020.594030

**Published:** 2021-01-08

**Authors:** Noëmi Rebecca Meier, Thomas M. Sutter, Marc Jacobsen, Tom H. M. Ottenhoff, Julia E. Vogt, Nicole Ritz

**Affiliations:** ^1^ Mycobacterial Research Laboratory, University of Basel Children’s Hospital, Basel, Switzerland; ^2^ Faculty of Medicine, University of Basel, Basel, Switzerland; ^3^ Department of Computer Science, Medical Data Science, Eidgenössische Technische Hochschule (ETH) Zurich, Zurich, Switzerland; ^4^ Department of General Pediatrics, Neonatology and Pediatric Cardiology, University Children's Hospital, Heinreich Heine University, Düsseldorf, Germany; ^5^ Department of Infectious Diseases, Leiden University Medical Center, Leiden, Netherlands; ^6^ Pediatric Infectious Diseases and Vaccinology Unit, University of Basel Children’s Hospital, Basel, Switzerland; ^7^ Department of Pediatrics, Royal Children’s Hospital Melbourne, University of Melbourne, Parkville, VIC, Australia

**Keywords:** cytokines, novel antigens, immune response, pediatric tuberculosis, interferon-gamma release assay

## Abstract

**Rationale:**

Tuberculosis diagnosis in children remains challenging. Microbiological confirmation of tuberculosis disease is often lacking, and standard immunodiagnostic including the tuberculin skin test and interferon-*γ* release assay for tuberculosis infection has limited sensitivity. Recent research suggests that inclusion of novel *Mycobacterium tuberculosis* antigens has the potential to improve standard immunodiagnostic tests for tuberculosis.

**Objective:**

To identify optimal antigen–cytokine combinations using novel *Mycobacterium tuberculosis* antigens and cytokine read-outs by machine learning algorithms to improve immunodiagnostic assays for tuberculosis.

**Methods:**

A total of 80 children undergoing investigation of tuberculosis were included (15 confirmed tuberculosis disease, five unconfirmed tuberculosis disease, 28 tuberculosis infection and 32 unlikely tuberculosis). Whole blood was stimulated with 10 novel *Mycobacterium tuberculosis* antigens and a fusion protein of early secretory antigenic target (ESAT)-6 and culture filtrate protein (CFP) 10. Cytokines were measured using xMAP multiplex assays. Machine learning algorithms defined a discriminative classifier with performance measured using area under the receiver operating characteristics.

**Measurements and main results:**

We found the following four antigen–cytokine pairs had a higher weight in the discriminative classifier compared to the standard ESAT-6/CFP-10-induced interferon-*γ*: Rv2346/47c- and Rv3614/15c-induced interferon-gamma inducible protein-10; Rv2031c-induced granulocyte-macrophage colony-stimulating factor and ESAT-6/CFP-10-induced tumor necrosis factor-α. A combination of the 10 best antigen–cytokine pairs resulted in area under the curve of 0.92 ± 0.04.

**Conclusion:**

We exploited the use of machine learning algorithms as a key tool to evaluate large immunological datasets. This identified several antigen–cytokine pairs with the potential to improve immunodiagnostic tests for tuberculosis in children.

## Introduction

Tuberculosis (TB) remains one of the leading causes of death globally. Current estimates show that one in ten TB cases occur in children below 15 years of age with an annual estimated number of one million cases of childhood TB disease in 2017 (World Health Organization, 2018a). Despite being a preventable and curable disease, 233,000 children died of TB in 2017, of which 80% occurred in children below 5 years of age. The recent World Health Organization roadmap towards ending TB in children and adolescents mentions up to 69% underdiagnosis and highlights the development of accurate, non-sputum-based diagnostics tests for TB disease and infection as a key action towards ending TB in children and adolescents (World Health Organization, 2018b).

TB infection is characterized by the absence of clinical signs and symptoms and evidence of containment of disease through the host immunological response. TB disease is usually defined as the active state of disease with loss of immunological containment, presence of symptoms and risk of transmission of disease. In young children TB disease is often of paucibacillary nature (*i.e.* low mycobacterial bacterial load) and therefore may remain undiagnosed using microbiological assays (Perez-Velez and Marais, 2012). In addition collection of samples for microbiological proof in this patient group is challenging and therefore TB confirmation reaches 50% at best (Oesch Nemeth et al., 2014). As a consequence, non-sputum-based diagnostic tests based on immunological evidence of TB have been developed. These tests rely on the measurement of a recall cell mediated immune response triggered by *in vivo* or *in vitro* mycobacterial antigens. Until two decades ago the tuberculin skin test has been the standard test, measuring a local skin induration after injection of purified protein derivative, a *Mycobacterium tuberculosis* protein mixture. However, because of its low specificity especially in Bacille Calmette–Guérin (TB vaccine prepared from an attenuated strain of *Mycobacterium bovis*) vaccinated individuals, interferon-gamma release assays have been developed, and have become the standard immunodiagnostic test of TB infection in adults (Diel et al., 2010). Interferon-gamma release assays are *in-vitro* blood-based assays measuring the *Mycobacterium tuberculosis*-specific immune response. Unfortunately these assays have two major limitations: lower performance in children with a sensitivity ranging from 62 to 83% and inability to discriminate between TB disease and TB infection (Mandalakas et al., 2011; Sollai et al., 2014). Recent research suggests that incorporation of novel *Mycobacterium tuberculosis* antigens expressed during different stages of TB [reviewed in (Meier et al., 2018)] and the measurement of additional cytokines (Walzl et al., 2011) can improve performance of currently used interferon-gamma release assay. Evaluation of novel diagnostic tests incorporating different *Mycobacterium tuberculosis* antigens and cytokines is therefore a feasible test suitable for pediatrics and urgently needed (World Health Organization, 2013).

The aim of our study was to include novel *Mycobacterium tuberculosis* antigens and measure additional cytokines for the immune diagnosis of childhood TB. We used supervised and unsupervised machine learning algorithms to compare groups and identify the best antigen–cytokine pairs.

## Methods

### Study Design, Setting, and Population

The Childhood Tuberculosis in Switzerland Study (CITRUS) is a prospective multicenter observational study (registered at ClinicalTrials.gov NCT03044509 and approved by the ethics committee EKNZ 2016-01094). In brief, eligible are children undergoing evaluation for TB exposure, infection or disease below the age of 18 years. Children that have been treated previously or that have started treatment more than 5 days before study inclusion are excluded. Upon enrolment baseline characteristics, clinical scores and TB test results done by the treating physician are recorded. The study participants were classified into the following groups confirmed TB, unconfirmed TB, TB infection, unlikely TB according to previously published case definitions (Graham et al., 2015) (for further details on study design and population see **Supplementary Methods Text**).

### Sample Preparation and Stimulation

**Figure 1:**
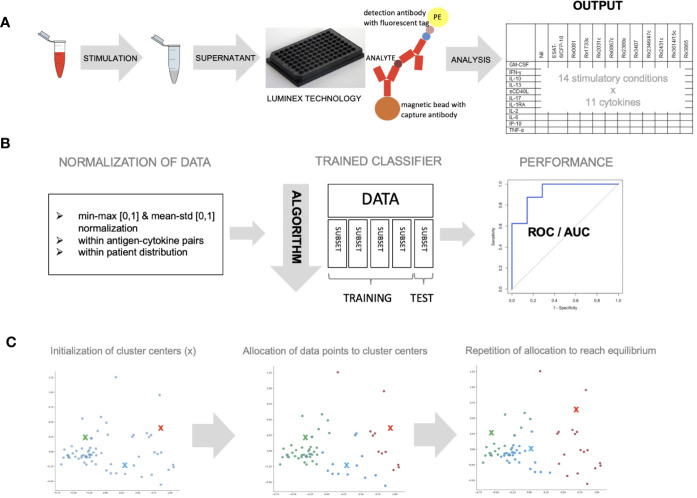
Whole blood was stimulated with novel antigens and data was analyzed with different machine learning algorithms. **(A)** Whole blood was stimulated with 11 mycobacterial antigens, left unstimulated and with a positive control, overnight and supernatant was analyzed using Luminex technology to measure 11 different cytokines **(B)** data was normalized within antigen–cytokine pairs using min-max or mean–std normalization or within patient distribution using the latter only. Data (n = 59) was divided into five equal parts and a classifier discriminating healthy *vs.* sick children was trained using four subsets and tested on one subset (cross-validation). The algorithm’s parameters were adjusted until performance was optimal. ROC curves were used to measure performance. **(C)** K-means clustering approach was used to allocate individual data points to three cluster centers randomly. This approach was repeated until optimal data point allocation was reached meaning the sum of the distances from data point to cluster centers is minimized.

Blood was collected in lithium-heparin tubes (Sarstedt Monovette 01.1608.100) and stimulated within 8 h of collection with 5 µg/ml phytohaemagglutinin (Merck chemicals LTD., Beeston, Nottingham, UK), 10 µg/ml staphylococcus enterotoxin B (Sigma Aldrich GmbH, Schnelldorf, Germany), 5 µg/ml of the following *Mycobacterium tuberculosis* recombinant proteins expressed and purified in *Escherichia coli* BL21: Rv0081, Rv1733c, Rv2031c, Rv0867c, Rv2389c, Rv3407, Rv2346/47c, Rv2431c, Rv3614/15c, Rv3865 and a fusion protein of early secretory antigenic target 6 (ESAT-6) and 10 kDa culture filtrate protein (CFP-10) [provided by the Department of Infectious Diseases at the University Leiden, the Netherlands (Franken et al., 2000),] and an unstimulated control (no protein added). The selection of the *Mycobacterium tuberculosis* recombinant proteins was based on published data summarized in a systematic literature review (Meier et al., 2018) and from unpublished data (personal communication THM Ottenhoff) CD28 and CD49d antibodies (Biolegend Inc., San Diego, Ca 92121, USA) were added at a concentration of 1 µg/ml to all conditions. Samples were stimulated overnight (16–18 h) at 37°C (**Figure 1A**).

### Cytokine Measurement

Granulocyte-macrophage colony-stimulating factor (GM-CSF), interferon (IFN)-*γ*, IFN-*γ*-inducible protein (IP)-10, interleukin (IL)-1 receptor-antagonist (RA), IL-2, IL-6, IL-10, IL-13, IL-17, soluble cluster of differentiation 40 ligand (sCD40L) and tumor necrosis factor (TNF)-α were measured using a Luminex technology according to the manufacturer’s instructions (**Figure 1A**, **Supplementary Methods Text**).

### Normalization of Data

Cytokine concentrations were normalized (Dodge, 2006) within antigen–cytokine pairs (using a minimum–maximum (min–max) or a mean-standard deviation (mean–std) normalization) and within a patient’s distribution of values (using a mean–std normalization) as indicated (**Figure 1B**).

### Discriminative Classifier

Discrimination of a pre-defined binary outcome (confirmed/unconfirmed TB and TB infection *versus* TB exposed), based on data containing information on all antigen–cytokine pairs (features), was achieved using a logistic regression classifier with L2-regularization (Hoerl and Kennard, 1970) (**Supplementary Methods Text**). To get a reliable estimate of the discriminative classifier performance, a five-fold cross-validation was applied to a set of training data to select the model’s hyperparameters (see **Supplementary Methods**). The performance of the discriminative classifier was evaluated using area under the receiver operating characteristics (AUROC) (Hanley and Mcneil, 1982). The contribution of each antigen–cytokine pair to our predictive model was evaluated by analyzing the weight in the decision function (**Figure 1B**).

### Unsupervised K-Means Clustering

K-means clustering algorithm (MacQueen, 1967) was performed with a predefined number of clusters (n = 3) reflecting the anticipated number of patient groups (confirmed/unconfirmed TB disease, TB infection, unlikely TB). Patients with incomplete measurements in any of the conditions (*e.g.* missing values) were excluded from this analysis. Cluster centers were allocated randomly at first, and every patient was then assigned to the nearest cluster center. Cluster center allocation and data point assignment were repeated until an equilibrium was reached (sum of distances is minimized, cluster centers not changed) (**Figure 1C**).

### Supervised K-Means Clustering Based on Median Cytokine Differences

Differences in median cytokine concentrations between confirmed/unconfirmed TB, TB infection and unlikely TB were compared. Antigen–cytokine pairs with the greatest differences were selected and K-means clustering approach was performed as above on these selected antigen–cytokine pairs.

## Results

A total of 80 patients were included: confirmed TB disease (n = 15), unconfirmed TB disease (n = 5), TB infection (n = 28), and unlikely TB (n = 32). Median age in the three TB groups was as follows: 9.7, 12.0, 11.3, and 5.8 years for confirmed TB, unconfirmed TB, TB infection, and unlikely TB (**Table 1**). A total of 49 of 80 (61.3%) children were tested for HIV, and all were negative. A total of 39 study participants out of 80 were born in Switzerland (48.8%), and 31 of 80 (38.8%) arrived in Switzerland less than 3 years prior to inclusion to the study. Routine immunodiagnostic testing was performed in 77 children with QuantiFERON-TB in 57/77 (74.0%) children, T-SPOT.TB in 10/77 (13.0%) and a tuberculin skin test in 40/77 (51.9%) children. Both interferon-gamma release assay and tuberculin skin test were done in 30 children and showed 23 (76.7%) concordant and 7 (23.3%) discordant results (one QuantiFERON-TB +/tuberculin skin test-; six QuantiFERON-TB −/tuberculin skin test+). Two T-SPOT.TB results were indeterminate (a confirmed TB disease case and an unconfirmed TB disease case).

**Table 1:** Baseline characteristics of the study population according to study group.

Variable	Confirmed TB	Unconfirmed TB	TB infection	Unlikely TB	Total
	N = 15	N = 5	N = 28	N = 32	N = 80
Median age, range (years)	9.7 (0.9–15.9)	12 (3–15.8)	11.3 (0.2–17.1)	5.8 (0.2–16.7)	9.6 (0.2–17.1)
IQR age	3.1–15.2 (12.1)	9.6–15.4 (5.8)	8.1–13.5 (5.4)	3.0–10.2 (7.2)	3.5–12.8 (9.3)
Males	6 (40%)	2 (40%)	15 (53.6%)	19 (59.4%)	42 (52.5%)
Median weight, range (m)	38.7 (11–75)	49 (13–60)	44 (10–71)	20 (8–65)	33.5 (8–75)
Ethnicity					
Caucasian	2	2	11	10	25
African	9	2	9	11	31
Asian	–	–	2	6	8
other	4	1	6	5	16
Country of birth					
Born in Switzerland	6	3	10	20	39
Recently migrated to Switzerland	9	2	11	9	31
Unknown	1	0	5	4	10
Symptoms					
Asymptomatic	5	1	25	29	60
Symptoms	10	4	3	2	19
cough	8	3	1	2	14
fever	6	2	0	2	10
unexplained fatigue	4	0	0	0	4
weight loss	4	2	0	0	6
lack of weight gain	1	0	0	0	1
other symptoms	3	4	1	1	9
Bacille Calmette-Guérin vaccination status					
vaccinated	4	0	9	8	21
not vaccinated	5	3	9	21	38
unknown	6	2	10	3	21
HIV status					
negative	12	5	14	18	49
positive	0	0	0	0	0
unknown	3	0	14	14	31
Tuberculin skin test					
not done	10	5	15	10	40
<5 mm	0	0	1	19	20
>5 mm	2	0	7	3	12
>15 mm	3	0	5	0	8
Imaging					
X ray	15	5	27	24	71
CT	8	4	3	1	16
compression	2	1	0	0	3
lymphadenopathy	9	2	0	0	11
consolidation parenchyma	11	4	0	2	17
miliary pattern	0	0	0	0	0
pleural effusion	3	0	0	0	3
cavitation	4	3	0	0	7
TSPOT					
not done	13	4	24	29	70
negative	0	0	0	3	3
positive	2	1	4	0	7
QuantiFERON-TB					
not done	5	2	5	11	23
negative	1	1	4	21	27
positive	9	2	19	0	30
Microbiological confirmation					
not done	0	0	22	30	52
culture positive	13	0	0	0	13
culture negative	0	4	6	2	12
PCR positive	13	0	0	0	13
PCR negative	0	5	4	0	9

### A Discriminative Classifier Distinguishes Healthy From Sick Children and Normalization of Data Results in Improvement of the Classifier’s Performance

**Figure 2:**
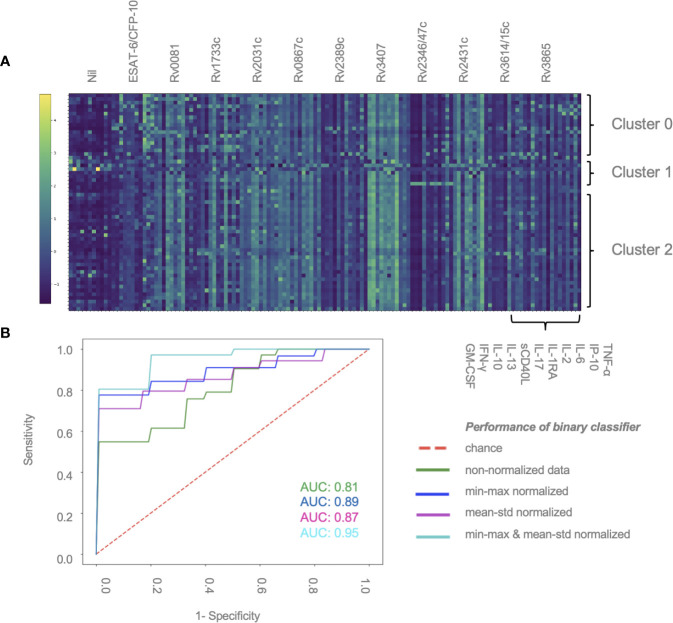
Normalization of data contributes to performance of discriminative classifier **(A)** Cytokine concentrations for individual patients. Results are sorted by patient group and clusters (2, 1 or 0), and antigen–cytokine pairs. Clustering was performed using K-means algorithm. Min–max normalization was applied to cytokine–antigen concentrations, mean–std normalization was applied to between-individual measurements (color change from dark blue to light green represents an increase in relative cytokine concentration). **(B)** AUROC curve showing the performance of the binary classifier (confirmed/unconfirmed TB and TB infection *versus* TB exposed) in 59 patients using different normalization methods: min–max and mean–std; normalization of antigen–cytokine pairs; min–max/mean-std combining an antigen–cytokine pair normalization with individual patient normalization.

**Figure 3:**
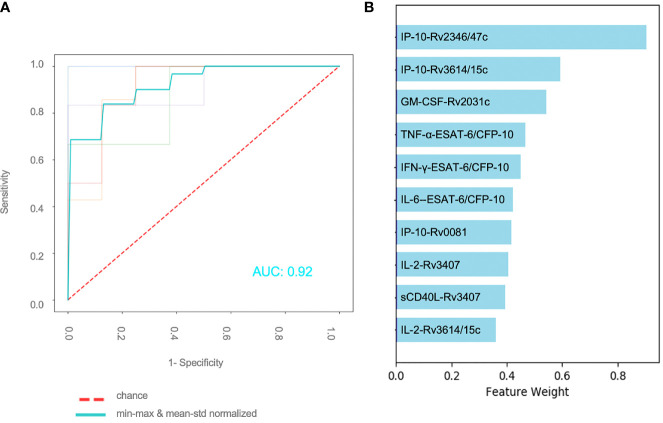
Effect of normalization of antigen–cytokine pairs and normalization for individual patients **(A)** Performance of binary classifier using the 10 most important features and applying an antigen–cytokine pair normalization (min–max) and a normalization for individual patients (mean–std) **(B)** Combination of 10 antigen–cytokine pairs contributing the most to performance of trained discriminative classifier with min–max normalization of antigen–cytokine pairs and mean–std individual patient normalization.

A total of 59 patients had complete measurements for all antigen–cytokine pairs and were included in this analysis: confirmed TB (n = 8), unconfirmed TB (n = 2), TB infection (n = 17) and unlikely TB (n = 32). Different methods of normalization (*e.g.* non-normalized data, antigen–cytokine pairs either normalized using min–max or mean–std normalization and normalization of antigen–cytokine pairs with min–max and between patient normalization with mean–std were applied to our dataset and resulted in differences on visual inspection of the graphs between antigen–cytokine pairs and cytokine concentrations (**Supplementary Figures S1A–D**). These differences influenced the outcome of the discriminative classifier (confirmed/unconfirmed TB and TB infection *versus* TB exposed). The AUROC was lower without normalization (AUROC = 0.81 ± 015), compared to a normalization of antigen–cytokine pairs (AUROC _min–max_ = 0.89 ± 0.12 and AUROC _mean–std_ = 0.87 ± 0.13) or combining an antigen–cytokine pair normalization with individual patient normalization (AUROC _min–max/mean–std_ = 0.95 ± 0.03) (**Figure 2B**). The most important antigen–cytokine pairs that contributed to the performance of the discriminative classifier were consistent for the normalization methods used. Rv2346/47c- and Rv3614/15c-induced concentrations of IP-10 were the two antigen–cytokine pairs with the highest weight in the predictive model for all discriminative classifiers with normalized data (**Figure 3B**, **Supplementary Figures S2A–C**). The weight of ESAT-6 and CFP-10-induced concentrations of TNF-α for the predictive model was consistently high for all normalized and non-normalized data. ESAT-6/CFP-10-induced concentrations of IFN-*γ* were among the 10 antigen–cytokine pairs that contributed the most to the classifier for all non-normalized and normalized data except when mean–std normalization alone was applied. Rv2031c-induced concentrations of GM-CSF contributed to the performance of the classifier when any normalization method was applied with increasing weight for combined min–max and mean–std normalization. Combining data from the 10 antigen–cytokine pairs with the highest weight in the predictive model using both min–max and mean–std normalization resulted in AUROC _min–max/mean–std_ = 0.92 ± 0.04 (**Figure 3A**).

### Unsupervised K-Means Clustering Reveals Three Groups of Children That Cannot Be Explained by Disease Status

K-means is a machine learning tool using vector quantization that groups observations into clusters based on distances to allocated cluster centers. Thereby we found three clusters which did not overlap with our patient groups (*i.e.* confirmed and unconfirmed TB, TB infection, unlikely TB) in the unsupervised analysis approach. All three clusters included patients from all study groups. **Figure 2A** displays normalized cytokine concentrations of antigen–cytokine pairs of all individual patients sorted by cluster (2, 1 or 0). Cluster 0 consisted of four confirmed TB, one unconfirmed TB, six TB infection and five unlikely TB patients (median age = 8.4, 68.7% male). Cluster 1 consisted of two confirmed TB, zero unconfirmed TB, two TB infection, and one unlikely TB patients (median age = 13.6, 20.0% male). Cluster 2 consisted of two confirmed TB, one unconfirmed TB, nine TB infection and 26 unlikely TB patients (median age = 7.8, 55.3% male). Clusters could neither be explained by disease classification, nor age, nor gender, nor ethnicity (data not shown).

### Supervised K-Means Clustering Based on Median Cytokine Differences Between Three Study Groups Reveals One Group That Clustered Mainly Healthy Children but No Confirmed TB Cases

Greatest differences in median cytokine concentrations between confirmed/unconfirmed TB, TB infection and unlikely TB were observed for: ESAT-6/CFP-10-induced concentrations of GM-CSF, IFN-*γ* and IL-2; Rv0081-induced concentrations of TNF-α; Rv2389c-induced concentrations of GM-CSF and IP-10; and Rv3614/15c-induced concentrations of IFN-*γ*, IL-2, IP-10 and TNF-α (data not shown). A total of 71 patients had complete measurements for these 10 conditions with the greatest differences and were thus further included in the comparative analysis: confirmed TB (n = 10), unconfirmed TB (n = 4), TB infection (n = 25) and unlikely TB (n = 32). K-means clustering with these antigen–cytokine pairs resulted in three cluster grouping the majority of unlikely TB patients and none of the confirmed TB patients in cluster 0 (25 out of 32). Only one unlikely TB patient and none of the unconfirmed TB patients were grouped to cluster 2 (six confirmed TB, five TB infection). Cluster 1 consisted of all four study groups with the majority being TB infected (11 out of 24) (**Supplementary Figures S3A–B**).

## Discussion

Diagnosis of childhood TB is one of the key challenges for the global epidemic. As current diagnostic tests are insufficient for detection of TB in children, there is an urgent need for novel tests. Our study is unique as it combines the use of the largest number of novel *Mycobacterium tuberculosis* antigens and cytokine combinations in a childhood TB diagnostic study, exploring the results by applying different machine learning algorithms.

We found that IP-10-responses induced by Rv2346/47c and Rv3614/15c were the two most important features to discriminate diseased from healthy individuals. We showed that further cytokines including GM-CSF, IL-2, IL-6, INF-γ and TNF-α play an important role during immune responses in TB in children. We also demonstrate the importance of data normalization to reduce bias towards highly expressed cytokines and inter-individual heterogeneity in *Mycobacterium tuberculosis*-specific immune responses.

Our selection of novel *Mycobacterium tuberculosis* antigens was based on previously published studies, and the antigens that are expressed during different stages of TB are briefly summarized below. The dormancy of survival regulon encoded antigens (Rv0081, Rv1733c, and Rv2031c) belong to a region of the *Mycobacterium tuberculosis* genome that includes approximately 50 genes associated with the non-replicative stage of TB (Voskuil et al., 2003). These antigens together with reactivation associated antigens (Rv0867c, Rv2389c, Rv3407) are highly immunogenic and have been tested mainly in adult cohorts [reviewed in (Meier et al., 2018)]. We also included the recently discovered *in vivo*-expressed antigens (Rv2346/47c, Rv2431c, Rv3614/15c, Rv3865) that have not been studied extensively in humans, but are believed to be important virulence factors (Commandeur et al., 2013). Rv2346 and Rv2347c are ESAT-6 like proteins and associated with downregulation of IL-6 and TNF-α enabling survival of bacteria inside macrophages (Malen et al., 2007; Yao et al., 2018). Rv2431c is a prolin-glutamic acid family protein, and its function is yet to be understood (Malen et al., 2007). Previous studies showed its involvement in necrosis in macrophages (Tundup et al., 2014) but also maturation and proliferation of dendritic cells (Chen et al., 2016). The antigens Rv3614c, Rv3615c and Rv3865 are all associated with the ESAT-6 secretion system 1 absent in the Bacille Calmette–Guérin vaccine strains.

The diagnostic potential of the recently discovered *in vivo*-expressed antigens found in our study has been shown in previous studies confirming our results (Millington et al., 2011). IFN-*γ* responses induced by Rv3615c were as specific as ESAT-6 and CFP-10 induced IFN-*γ* responses in patients with TB disease and infection (Millington et al., 2011). The antigen Rv3615c was included in a modified T-SPOT.TB assay and was shown to improve the diagnosis of TB disease and infection compared to healthy controls and patients with non-TB lung disease (Li et al., 2017). The use of Rv3865 seems to be of limited value also shown by the low immunogenic potential in other studies in adults (Bahk et al., 2004) and adolescents including different stages of TB infection (Michelsen et al., 2017).

In our study we found dormancy of survival regulon encoded antigens to be of key importance eliciting a differential immune response in TB patients and exposed healthy controls. We found that the dormancy of survival regulon antigens Rv0081 and Rv2031c-induced IP-10 and GM-CSF responses contributed strongly to performance of the discriminative classifier. Several studies in adults reported elevated concentrations of cytokines induced by Rv0081 during TB infection and disease, which is in line with our findings [reviewed in (Meier et al., 2018)]. In contrast to our findings, studies in adults suggest Rv1733c-induced immune responses to be of added diagnostic value (Leyten et al., 2006; Kassa et al., 2012; Mensah et al., 2014; Serra-Vidal et al., 2014). Furthermore, previous studies including Rv2031c-induced cytokine response, showed conflicting results with one study reporting higher concentrations of IFN-*γ*, IL-10, and TNF-α in TB exposed individuals compared to healthy controls (Belay et al., 2015) and other studies failing to show IFN-*γ* responses induced by this antigen (Goletti et al., 2010; Hozumi et al., 2013). Our study supports the notion that Rv2031c-induced responses are important as diagnostic markers for TB particularly when cytokines other than IFN-*γ* are included into the analysis. This is in line with Coppola et al. showing high concentrations of TNF-α expression in response to Rv2031c in addition to other cytokines such as IP-10 or IL-17 but notably not IFN-*γ* (Coppola et al., 2016).

In addition to the above, two reactivation-associated antigens were found to be important in our study: Rv3407 and Rv2389c. We found that Rv2389c-induced GM-CSF and IP-10 responses were among the 10 antigen–cytokine pairs that contributed the most to discriminating between sick and healthy. Other studies also show the diagnostic potential of Rv2389c. IFN-*γ* responses induced by Rv0867c and Rv2389c were found to be higher in individuals with TB infection compared to healthy controls and TB disease in several studies (Commandeur et al., 2011; Chegou et al., 2012; Serra-Vidal et al., 2014). High concentrations of IL-6, IL-10, and TNF-α were found to be induced by Rv0867c and Rv2389c in individuals with TB disease (Kassa et al., 2012). In our study, however, Rv0867c did not induce cytokine responses that contributed to classification of patients.

The standard antigens used in the current available test including ESAT-6 and CFP-10 remain important. Our results, however, clearly show that in addition to IFN-*γ* also IL-6 and TNF-α responses to ESAT-6 and CFP-10 contributed towards distinction of study groups and were among the 10 most important features for the discriminative classifier. Two studies in children also confirm the addition of TNF-α to improve distinction between TB patients and healthy individuals (Tebruegge et al., 2015; Tebruegge et al., 2019).

For the read-out of antigen stimulated-blood it has been shown in numerous studies that cytokines other than IFN-*γ* play an important role during the course of infection and may therefore have added diagnostic value (Kassa et al., 2012; Chegou et al., 2012; Belay et al., 2015; Coppola et al., 2016; Tebruegge et al., 2019). A selection of pro- and anti-inflammatory cytokines was therefore included in our study on the basis of previously published research (Walzl et al., 2011; Meier et al., 2018). Our findings suggest that measuring IFN-*γ* only has limited diagnostic potential and that measurement of other cytokines has clear added diagnostic value. In particular, IP-10—a chemokine produced by antigen-presenting cells and induced by a large number of cytokines including IFN-α, IFN-*β*, IFN-*γ*, IL-1β, IL-2, IL-17, IL-23, TNF-α (Hassanshahi et al., 2007; Mohty et al., 2010)—has been shown to be important in previous studies and our current study. In our study IP-10 concentrations were generally high for all antigens, which were also noted in earlier studies in children (Latorre et al., 2014; Jenum et al., 2016; Petrone et al., 2018). The high measurable concentrations of this cytokine may improve robustness of immunodiagnostic tests especially in children and immunocompromised individuals (Ruhwald et al., 2012). Several studies in adults have shown elevated IP-10 responses in TB disease patients compared to controls (Chegou et al., 2009; Kabeer et al., 2010; Ruhwald et al., 2011). Furthermore antigen-induced IP-10 concentrations were higher in TB disease patients and children from high endemic countries and high-risk groups (Ruhwald et al., 2008; Lighter et al., 2009). One further important aspect particularly interesting for studies in children is the fact that several previous studies suggest IP-10 may be less affected by age as compared to IFN-*γ* (Lighter et al., 2009; Lighter-Fisher et al., 2010). By contrast there are some studies that did find an age-association for IP-10 concentrations (Ruhwald et al., 2008; Decker et al., 2017). Earlier work from our group in healthy children only found an age-association for *Candida albicans*-induced IP-10 concentrations but not for other stimuli (Decker et al., 2017). GM-CSF is thought to have a protective role in the control of TB infection. In our study latency associated antigen Rv2031c induced differential GM-CSF response in healthy and sick individuals. Studies in mice show that deficiency in GM-CSF results in the inability to contain infection (Gonzalez-Juarrero et al., 2005). Other research suggests that survival of bacteria in macrophages is regulated by GM-CSF response in macrophages (Bryson et al., 2019).

In our study we demonstrate the impact of normalization on data with improved performance of a discriminative classifier. Performance was best and most robust when both cytokine-antigen concentrations and between-patient values were normalized. IP-10 concentrations induced by Rv2346/47c and Rv3614/15c were found as major contributors to the performance of the discriminative classifier throughout all normalization methods, likely resulting from high concentrations of this cytokine. However, for cytokines that are not expressed at high concentrations, we show that normalization is highly important. For example, IL-2 and IFN-*γ* concentrations induced by ESAT-6/CFP-10 and Rv3614/15c were only shown to be among the most important features after normalization.

One potential limitation of our study is the sample size which was limited for the two subgroups of TB infection and disease. For optimal training of the classifier and differentiation between TB infection and disease a larger sample size is required. Further studies including a larger number of children are therefore needed to confirm and expand our results. In addition, this study is conducted in a low incidence setting and major factors influencing immune responses such as malnutrition, HIV-infection and other immunocompromising conditions are rare and can therefore not be evaluated.

In conclusion, this is the first study using machine learning algorithms to analyze results from novel *Mycobacterium tuberculosis* antigens and cytokines for the immunodiagnosis of TB in children. The use of machine learning algorithms is a key tool to evaluate large immunological datasets. We identified antigen–cytokine pairs that perform better than the current standard antigen–cytokine pair used in interferon-gamma release assays. These results show that novel antigen–cytokine pairs have to potential to improve immunodiagnostic tests for tuberculosis in children.

## Data Availability Statement

The raw data supporting the conclusions of this article will be made available by the authors, without undue reservation.

## Ethics Statement

The studies involving human participants were reviewed and approved by Ethikkommission Nordwestschweiz. Written informed consent to participate in this study was provided by the participants’ legal guardian/next of kin.

## Members of the CITRUS study team

Andrea Duppenthaler, Anne Mornand, Christa Relly, Christian Kahlert, Christoph Berger, Isabelle Rochat Guignard, Jürg Barben, Deborah Levet, Lisa Kottanattu, Marie Rohr, Michael Buettcher, Sara Bernhard-Stirnemann and Nicole Ritz.

## Author Contributions

NM and NR developed the research question and the study design. NM performed the experiments. TS, NM, JV, and NR performed the data analysis. NM and NR wrote the draft manuscript. All authors contributed to the article and approved the submitted version.

## Funding

NM was supported by the following associations: Bangerter Rhyner Stiftung, Lunge Zürich, Nora van Meeuwen-Häfliger Stiftung, Rozalia Foundation, Schweizerische Lungenstiftung and Nikolaus and Bertha Burckhardt Bürgin Foundation.

## Conflict of Interest

The authors declare that the research was conducted in the absence of any commercial or financial relationships that could be construed as a potential conflict of interest.

## Supplementary Material

Supplementary Figure 1 relative median cytokine concentrations, (color change from dark blue to light green indicates an increase in relative cytokine concentration). Non-normalized data **(A)**, min–max normalized data **(B)**, mean–std normalized data **(C)**, min–max normalized and mean–std normalized (between individuals) data **(D)**.

Supplementary Figure 2 Normalization of data contributes to the performance of a discriminative classifier. Combination of 10 antigen–cytokine pairs contributing the most to the performance of a trained discriminative classifier according to different normalization methods applied: **(A)** non-normalized data **(B)** min–max normalized data and **(C)** mean–std normalized data.

Supplementary Figure 3 Normalized cytokine concentrations for individual patients (n = 71) and selected antigen–cytokine pairs sorted by clusters **(A)** and study group **(B)** (color change from dark blue to light green indicates an increase in relative cytokine concentration.

## Abbreviations

AUROC, area under the receiver operating characteristic; CFP-10, 10 kDA culture filtrate protein; CITRUS, Childhood Tuberculosis in Switzerland Study; ESAT-6, early secretory antigenic target 6; GM-CSF, granulocyte-macrophage colony-stimulating factor; IFN, interferon; IL, interleukin; IP-10, interferon-gamma inducible protein 10; min–max, minimum–maximum; mean–std, mean – standard deviation; sCD40L, soluble cluster of differentiation 40 ligand; TB, tuberculosis; TNF, tumor necrosis factor.

## References

[B1] Bahk Y. Y. Kim S. A. Kim J. S. Euh H. J. Bai G. H. Cho S. N. (2004). Antigens secreted from Mycobacterium tuberculosis: identification by proteomics approach and test for diagnostic marker. Proteomics 4 (11), 3299–3307. 10.1002/pmic.200400980 15378731

[B2] Belay M. Legesse M. Mihret A. Bekele Y. Ottenhoff T. H. Franken K. L. (2015). Pro- and anti-inflammatory cytokines against Rv2031 are elevated during latent tuberculosis: a study in cohorts of tuberculosis patients, household contacts and community controls in an endemic setting. PloS One 10 (4), e0124134. 10.1371/journal.pone.0124134 25897840 PMC4405476

[B3] Bryson B. D. Rosebrock T. R. Tafesse F. G. Itoh C. Y. Nibasumba A. Babunovic G. H. (2019). Heterogeneous GM-CSF signaling in macrophages is associated with control of Mycobacterium tuberculosis. Nat. Commun. 10 (1), 2329. 10.1038/s41467-019-10065-8 31133636 PMC6536549

[B4] Chegou N. N. Black G. F. Kidd M. van Helden P. D. Walzl G. (2009). Host markers in QuantiFERON supernatants differentiate active TB from latent TB infection: preliminary report. BMC Pulm. Med. 9, 21. 10.1186/1471-2466-9-21 19445695 PMC2696407

[B5] Chegou N. N. Essone P. N. Loxton A. G. Stanley K. Black G. F. van der Spuy G. D. (2012). Potential of host markers produced by infection phase-dependent antigen-stimulated cells for the diagnosis of tuberculosis in a highly endemic area. PloS One 7 (6), e38501. 10.1371/annotation/bc36a9c6-d5c0-4d55-bc92-9ce4a07b4f70 22693640 PMC3367928

[B6] Chen W. Bao Y. Chen X. Burton J. Gong X. Gu D. (2016). Mycobacterium tuberculosis PE25/PPE41 protein complex induces activation and maturation of dendritic cells and drives Th2-biased immune responses. Med. Microbiol. Immunol. 205 (2), 119–131. 10.1007/s00430-015-0434-x 26318856

[B7] Commandeur S. van Meijgaarden K. E. Lin M. Y. Franken K. L. Friggen A. H. Drijfhout J. W. (2011). Identification of human T-cell responses to Mycobacterium tuberculosis resuscitation-promoting factors in long-term latently infected individuals. Clin. Vaccine Immunol. 18 (4), 676–683. 10.1128/CVI.00492-10 21248154 PMC3122556

[B8] Commandeur S. van Meijgaarden K. E. Prins C. Pichugin A. V. Dijkman K. van den Eeden S. J. (2013). An unbiased genome-wide Mycobacterium tuberculosis gene expression approach to discover antigens targeted by human T cells expressed during pulmonary infection. J. Immunol. 190 (4), 1659–1671. 10.4049/jimmunol.1201593 23319735

[B9] Coppola M. van Meijgaarden K. E. Franken K. L. Commandeur S. Dolganov G. Kramnik I. (2016). New Genome-Wide Algorithm Identifies Novel In-Vivo Expressed Mycobacterium Tuberculosis Antigens Inducing Human T-Cell Responses with Classical and Unconventional Cytokine Profiles. Sci. Rep. 6, 37793. 10.1038/srep37793 27892960 PMC5125271

[B10] Decker M. L. Gotta V. Wellmann S. Ritz N. (2017). Cytokine profiling in healthy children shows association of age with cytokine concentrations. Sci. Rep. 7 (1), 17842. 10.1038/s41598-017-17865-2 29259216 PMC5736560

[B11] Diel R. Loddenkemper R. Nienhaus A. (2010). Evidence-based comparison of commercial interferon-gamma release assays for detecting active TB: a metaanalysis. Chest 137 (4), 952–968. 10.1378/chest.09-2350 20022968

[B12] Dodge Y. (2006). The Oxford Dictionary of Statistical Terms Ed. Y Dodge (Oxford: Oxford University Press).

[B13] Franken K. L. Hiemstra H. S. van Meijgaarden K. E. Subronto Y. den Hartigh J. Ottenhoff T. H. (2000). Purification of his-tagged proteins by immobilized chelate affinity chromatography: the benefits from the use of organic solvent. Protein Expr. Purif. 18 (1), 95–99. 10.1006/prep.1999.1162 10648174

[B14] Goletti D. Butera O. Vanini V. Lauria F. N. Lange C. Franken K. L. (2010). Response to Rv2628 latency antigen associates with cured tuberculosis and remote infection. Eur. Respir. J. 36 (1), 135–142. 10.1183/09031936.00140009 19926735

[B15] Gonzalez-Juarrero M. Hattle J. M. Izzo A. Junqueira-Kipnis A. P. Shim T. S. Trapnell B. C. (2005). Disruption of granulocyte macrophage-colony stimulating factor production in the lungs severely affects the ability of mice to control Mycobacterium tuberculosis infection. J. Leukoc. Biol. 77 (6), 914–922. 10.1189/jlb.1204723 15767289

[B16] Graham S. M. Cuevas L. E. Jean-Philippe P. Browning R. Casenghi M. Detjen A. K. (2015). Clinical Case Definitions for Classification of Intrathoracic Tuberculosis in Children: An Update. Clin. Infect. Dis. 61Suppl 3, S179–S187. 10.1093/cid/civ581 PMC4583568 26409281

[B17] Hanley J. A. Mcneil B. J. (1982). The Meaning and Use of the Area Under a Receiver Operating Characterstics (ROC) Curve. Radiology 143 (1), 29–36. 10.1148/radiology.143.1.7063747 7063747

[B18] Hassanshahi G. Jafarzadeh A. Ghorashi Z. Zia Sheikholeslami N. Dickson A. J. (2007). Expression of IP-10 chemokine is regulated by pro-inflammatory cytokines in cultured hepatocytes. Iran. J. Allergy Asthma Immunol. 6 (3), 115–121. 17893431

[B19] Hoerl A. E. Kennard R. W. (1970). Ridge Regression: Biased Estimation for Nonorthogonal Problems. Technometrics 12 (1), 55–67. 10.1080/00401706.1970.10488634

[B20] Hozumi H. Tsujimura K. Yamamura Y. Seto S. Uchijima M. Nagata T. (2013). Immunogenicity of dormancy-related antigens in individuals infected with Mycobacterium tuberculosis in Japan. Int. J. Tuberc. Lung Dis. 17 (6), 818–824. 10.5588/ijtld.12.0695 23676169

[B21] Jenum S. Dhanasekaran S. Ritz C. Macaden R. Doherty T. M. Grewal H. M. S. (2016). Added Value of IP-10 as a Read-Out of Mycobacterium tuberculosis Specific Immunity in Young Children. Pediatr. Infect. Dis. J. 35 (12), 1336–1338. 10.1097/INF.0000000000001328 27642776 PMC5108305

[B22] Kabeer B. S. A. Raman B. Thomas A. Perumal V. Raja A. (2010). Role of QuantiFERON-TB Gold, Interferon Gamma Inducible Protein-10 and Tuberculin Skin Test in Active Tuberculosis Diagnosis. PloS One 5 (2). 10.1371/journal.pone.0009051 PMC2816212 20140219

[B23] Kassa D. Ran L. Geberemeskel W. Tebeje M. Alemu A. Selase A. (2012). Analysis of immune responses against a wide range of Mycobacterium tuberculosis antigens in patients with active pulmonary tuberculosis. Clin. Vaccine Immunol. 19 (12), 1907–1915. 10.1128/CVI.00482-12 23015647 PMC3535869

[B24] Latorre I. Díaz J. Mialdea I. Serra-Vidal M. Altet N. Prat C. (2014). IP-10 is an accurate biomarker for the diagnosis of tuberculosis in children. J. Infect. 69 (6), 590–599. 10.1016/j.jinf.2014.06.013 24975172

[B25] Leyten E. M. Lin M. Y. Franken K. L. Friggen A. H. Prins C. van Meijgaarden K. E. (2006). Human T-cell responses to 25 novel antigens encoded by genes of the dormancy regulon of Mycobacterium tuberculosis. Microbes Infect. 8 (8), 2052–2060. 10.1016/j.micinf.2006.03.018 16931093

[B26] Li G. Li F. Zhao H. M. Wen H. L. Li H. C. Li C. L. (2017). Evaluation of a New IFN-gamma Release Assay for Rapid Diagnosis of Active Tuberculosis in a High-Incidence Setting. Front. Cell. Infect. Microbiol. 7, 117. 10.3389/fcimb.2017.00117 28443247 PMC5386965

[B27] Lighter J. Rigaud M. Huie M. Peng C. H. Pollack H. (2009). Chemokine IP-10: an adjunct marker for latent tuberculosis infection in children. Int. J. Tuberculosis Lung Dis. 13 (6), 731–736. 19460249

[B28] Lighter-Fisher J. Peng C. H. Tse D. B. (2010). Cytokine responses to QuantiFERON® peptides, purified protein derivative and recombinant ESAT-6 in children with tuberculosis. Int. J. Tuberculosis Lung Dis. 14 (12), 1548–1555. 21144239

[B29] MacQueen J. (1967). Some methods for classification and analysis of multivariate observations. Proceedings of the 5th Berkeley Symposium on Mathematical Statistics and Probability, Vol. 1) (Berkeley, Calif.: University of California Press). p.281–p.297. Available at: https://projecteuclid.org/euclid.bsmsp/1200512992.

[B30] Malen H. Berven F. S. Fladmark K. E. Wiker H. G. (2007). Comprehensive analysis of exported proteins from Mycobacterium tuberculosis H37Rv. Proteomics 7 (10), 1702–1718. 10.1002/pmic.200600853 17443846

[B31] Mandalakas A. M. Detjen A. K. Hesseling A. C. Benedetti A. Menzies D. (2011). Interferon-gamma release assays and childhood tuberculosis: systematic review and meta-analysis. Int. J. Tuberc. Lung Dis. 15 (8), 1018–1032. 10.5588/ijtld.10.0631 21669030

[B32] Meier N. R. Jacobsen M. Ottenhoff T. H. M. Ritz N. (2018). A Systematic Review on Novel Mycobacterium tuberculosis Antigens and Their Discriminatory Potential for the Diagnosis of Latent and Active Tuberculosis. Front. Immunol. 9, 2476. 10.3389/fimmu.2018.02476 30473692 PMC6237970

[B33] Mensah G. I. Addo K. K. Tetteh J. A. Sowah S. Loescher T. Geldmacher C. (2014). Cytokine response to selected MTB antigens in Ghanaian TB patients, before and at 2 weeks of anti-TB therapy is characterized by high expression of IFN-gamma and Granzyme B and inter- individual variation. BMC Infect. Dis. 14, 495. 10.1186/1471-2334-14-495 25209422 PMC4180837

[B34] Michelsen S. W. Soborg B. Diaz L. J. Hoff S. T. Agger E. M. Koch A. (2017). The dynamics of immune responses to Mycobacterium tuberculosis during different stages of natural infection: A longitudinal study among Greenlanders. PloS One 12 (6), e0177906. 10.1371/journal.pone.0177906 28570574 PMC5453477

[B35] Millington K. A. Fortune S. M. Low J. Garces A. Hingley-Wilson S. M. Wickremasinghe M. (2011). Rv3615c is a highly immunodominant RD1 (Region of Difference 1)-dependent secreted antigen specific for Mycobacterium tuberculosis infection. Proc. Natl. Acad. Sci. U. S. A. 108 (14), 5730–5735. 10.1073/pnas.1015153108 21427227 PMC3078386

[B36] Mohty A. M. Grob J. J. Mohty M. Richard M. A. Olive D. Gaugler B. (2010). Induction of IP-10/CXCL10 secretion as an immunomodulatory effect of low-dose adjuvant interferon-alpha during treatment of melanoma. Immunobiology 215 (2), 113–123. 10.1016/j.imbio.2009.03.008 19450896

[B37] Oesch Nemeth G. Nemeth J. Altpeter E. Ritz N. (2014). Epidemiology of childhood tuberculosis in Switzerland between 1996 and 2011. Eur. J. Pediatr. 173 (4), 457–462. 10.1007/s00431-013-2196-z 24202411

[B38] Perez-Velez C. M. Marais B. J. (2012). Tuberculosis in children. N. Engl. J. Med. 367 (4), 348–361. 10.1056/NEJMra1008049 22830465

[B39] Petrone L. Vanini V. Chiacchio T. Petruccioli E. Cuzzi G. Schinina V. (2018). Evaluation of IP-10 in Quantiferon-Plus as biomarker for the diagnosis of latent tuberculosis infection. Tuberculosis 111, 147–153. 10.1016/j.tube.2018.06.005 30029901

[B40] Ruhwald M. Petersen J. Kofoed K. Nakaoka H. Cuevas L. E. Lawson L. (2008). Improving T-Cell Assays for the Diagnosis of Latent TB Infection: Potential of a Diagnostic Test Based on IP-10. PloS One 3 (8). 10.1371/journal.pone.0002858 PMC2483344 18682747

[B41] Ruhwald M. Dominguez J. Latorre I. Losi M. Richeldi L. Pasticci M. B. (2011). A multicentre evaluation of the accuracy and performance of IP-10 for the diagnosis of infection with M. tuberculosis. Tuberculosis 91 (3), 260–267. 10.1016/j.tube.2011.01.001 21459676

[B42] Ruhwald M. Aabye M. G. Ravn P. (2012). IP-10 release assays in the diagnosis of tuberculosis infection: current status and future directions. Expert Rev. Mol. Diagn. 12 (2), 175–187. 10.1586/erm.11.97 22369377

[B43] Serra-Vidal M. M. Latorre I. Franken K. L. Diaz J. de Souza-Galvao M. L. Casas I. (2014). Immunogenicity of 60 novel latency-related antigens of Mycobacterium tuberculosis. Front. Microbiol. 5, 517. 10.3389/fmicb.2014.00517 25339944 PMC4189613

[B44] Sollai S. Galli L. de Martino M. Chiappini E. (2014). Systematic review and meta-analysis on the utility of Interferon-gamma release assays for the diagnosis of Mycobacterium tuberculosis infection in children: a 2013 update. BMC Infect. Dis. 14. 10.1186/1471-2334-14-S1-S6 PMC4016555 24564486

[B45] Tebruegge M. Dutta B. Donath S. Ritz N. Forbes B. Camacho-Badilla K. (2015). Mycobacteria-Specific Cytokine Responses Detect Tuberculosis Infection and Distinguish Latent from Active Tuberculosis. Am. J. Respir. Crit. Care Med. 192 (4), 485–499. 10.1164/rccm.201501-0059OC 26030187

[B46] Tebruegge M. Ritz N. Donath S. Dutta B. Forbes B. Clifford V. (2019). Mycobacteria-Specific Mono- and Polyfunctional CD4+ T Cell Profiles in Children With Latent and Active Tuberculosis: A Prospective Proof-of-Concept Study. Front. Immunol. 10, 431. 10.3389/fimmu.2019.00431 31024518 PMC6459895

[B47] Tundup S. Mohareer K. Hasnain S. E. (2014). Mycobacterium tuberculosis PE25/PPE41 protein complex induces necrosis in macrophages: Role in virulence and disease reactivation? FEBS Open Bio 4, 822–828. 10.1016/j.fob.2014.09.001 PMC4219985 25379378

[B48] Voskuil M. I. Schnappinger D. Visconti K. C. Harrell M. I. Dolganov G. M. Sherman D. R. (2003). Inhibition of respiration by nitric oxide induces a Mycobacterium tuberculosis dormancy program. J. Exp. Med. 198 (5), 705–713. 10.1084/jem.20030205 12953092 PMC2194188

[B49] Walzl G. Ronacher K. Hanekom W. Scriba T. J. Zumla A. (2011). Immunological biomarkers of tuberculosis. Nat. Rev. Immunol. 11 (5), 343–354. 10.1038/nri2960 21475309

[B50] World Health Organization (2013). Roadmap for childhood tuberculosis. Geneva; WHO.

[B51] World Health Organization (2018a). Global Tuberculosis Report 2018. Geneva; WHO.

[B52] World Health Organization (2018b). Roadmap towards ending TB in children and adolescents. Geneva; WHO.

[B53] Yao J. Du X. Chen S. Shao Y. Deng K. Jiang M. (2018). Rv2346c enhances mycobacterial survival within macrophages by inhibiting TNF-alpha and IL-6 production via the p38/miRNA/NF-kappaB pathway. Emerg. Microbes Infect. 7 (1), 158. 10.1038/s41426-018-0162-6 30232332 PMC6145905

