# Supplementary figures and images for: Machine Learning Algorithms Evaluate Immune Response to Novel Mycobacterium tuberculosis Antigens for Diagnosis of Tuberculosis
Source: Front Cell Infect Microbiol. 2021 Jan 8;10:594030. doi: 10.3389/fcimb.2020.594030 (PMC7820115; doi:10.3389/fcimb.2020.594030)

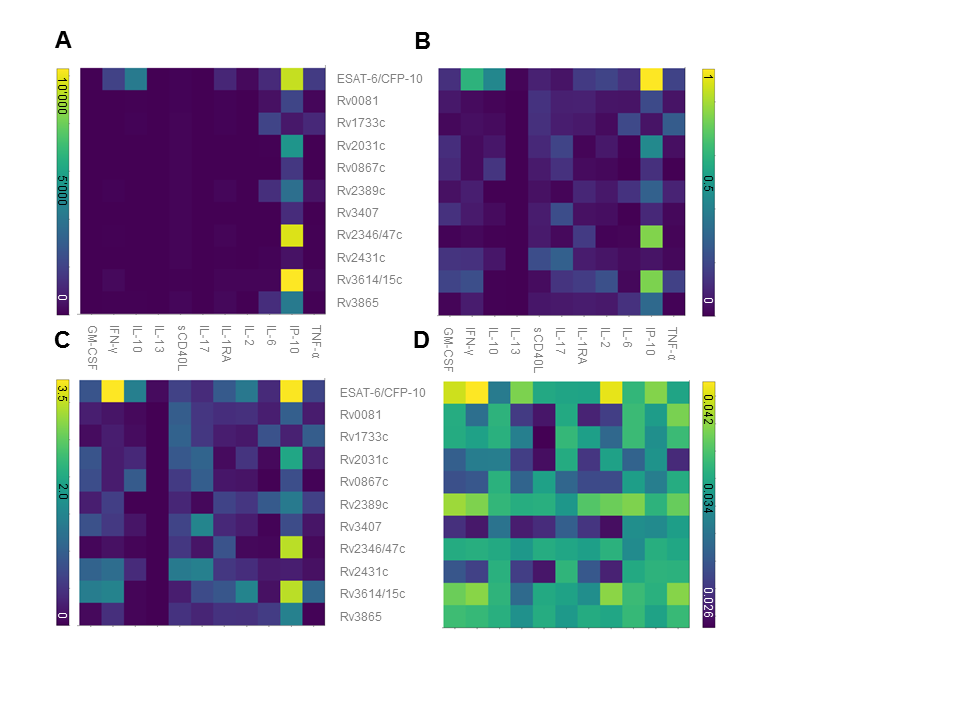

Supplement: Supplementary Figure 1 — relative median cytokine concentrations, (color change from dark blue to light green indicates an increase in relative cytokine concentration). Non-normalized data (A), min–max normalized data (B), mean–std normalized data (C), min–max normalized and mean–std normalized (between individuals) data (D). [file Image_1.tif]

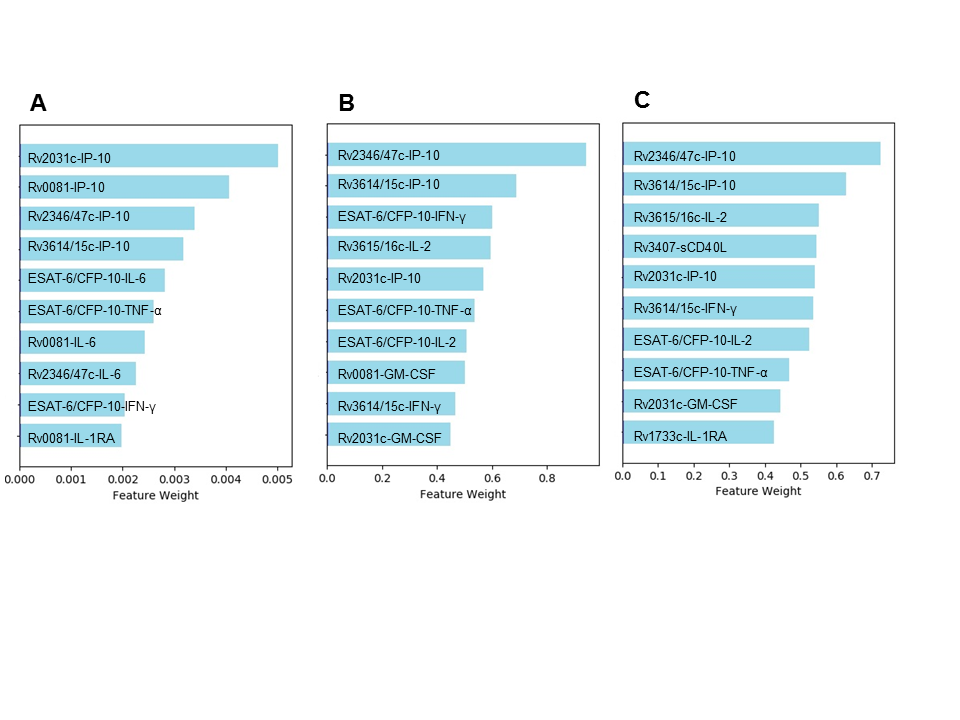

Supplement: Supplementary Figure 2 — Normalization of data contributes to the performance of a discriminative classifier. Combination of 10 antigen–cytokine pairs contributing the most to the performance of a trained discriminative classifier according to different normalization methods applied: (A) non-normalized data (B) min–max normalized data and (C) mean–std normalized data. [file Image_2.tif]

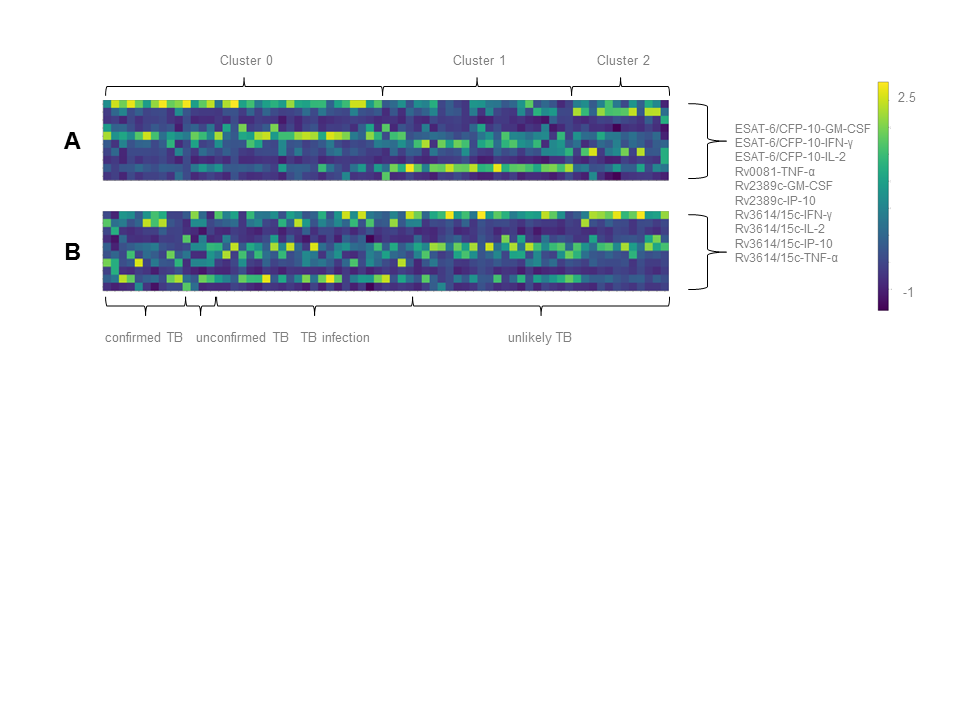

Supplement: Supplementary Figure 3 — Normalized cytokine concentrations for individual patients (n = 71) and selected antigen–cytokine pairs sorted by clusters (A) and study group (B) (color change from dark blue to light green indicates an increase in relative cytokine concentration. [file Image_3.tif]
