# Supplementary material for: Machine Learning Algorithms Evaluate Immune Response to Novel Mycobacterium tuberculosis Antigens for Diagnosis of Tuberculosis
Source: Front Cell Infect Microbiol. 2021 Jan 8;10:594030. doi: 10.3389/fcimb.2020.594030 (PMC7820115; doi:10.3389/fcimb.2020.594030)
Supplement: Supplementary file 4 [file DataSheet_1.docx]

**Supplementary methods**

**Study design, setting and population.** The CITRUS study is registered at ClinicalTrials.gov (NCT03044509) and was approved by the ethics committees of all different study centers and written consent was obtained from all participants and/or their legal representatives (lead ethics Ethikkommission Nordwest- und Zentralschweiz 2016-01094). Baseline characteristics recorded upon enrollment include information on country of birth, age, ethnicity, Bacille Calmette-Guérin immunization status, nutritional status, medical history. Participants are managed according to current Swiss guidelines by the local treating team. The study participants were classified into the following groups confirmed TB (microbiologically confirmed TB disease using culture or nucleic acid amplification techniques), unconfirmed TB (no microbiological proof of TB but symptoms and/or a chest radiograph suggestive of TB and/or routine immunologic evidence of TB by TST (>5mm) or positive IGRA), TB infection (criteria for confirmed and unconfirmed TB not met but routine immunologic evidence of TB by TST (>5mm) or positive IGRA), unlikely TB (exposed healthy children that did not meet the above criteria and had no immunologic evidence of TB by routine TST (≤ 5mm) or negative IGRA) according to previously published case definitions by Graham et al.

**Cytokine measurement**. Cytokine concentrations were calculated using a standard curve (5-parameter logistic regression). Concentrations of unstimulated samples served as background and were subtracted from antigen- and mitogen-induced cytokine concentrations. Measurements above or below the limit of quantification (calibration range: 3.2-10’000 pg/ml) were set to 0.1 pg/ml for the lower and to 10’000 pg/ml for the upper limit of quantification.

**Normalization of data.** A min-max normalization, called min-max feature scaling, was applied to the dataset by mapping the entire range of cytokine concentrations to the range [0,1] for every pair. A mean-std normalization, was applied to the dataset by transforming the distribution of cytokine concentrations of every patient to have a mean of zero and a standard deviation of 1.

**Discriminative classifier.** L2-regularisation: By adding the L2 norm of the coefficients to the loss function, we reduce the model complexity as the regularization term enforces a shrinking of the coefficients. Large weights in the model are penalized, which helps in reducing overfitting.

Five-fold cross-validation: the dataset was split into five equally sized parts, for the model to be trained on four parts and tested on one. This was repeated for all combinations of subsets for a single set of hyperparameters to report a more stable and reliable estimate of the model’s predictive power.

All data was analyzed using Python (version 3.6) including the python libraries Pandas (version 0.24), Scikit-learn (version 0.21) and Numpy (version 1.16). The plots were generated using the Python library Matplotlib (version 3.0)
